# Contextualized Experiences and Predictors of Condom Use in a Flemish Population: A Mixed Methods Study

**DOI:** 10.3390/ijerph21121545

**Published:** 2024-11-21

**Authors:** Alexis Dewaele, Eva Koppen, Sandra Van den Eynde

**Affiliations:** 1 Department of Experimental Clinical and Health Psychology, Faculty of Psychology and Educational Sciences, Ghent University, Henri Dunantlaan 2, 9000 Gent, Belgium; 2 Sensoa, Flemish Expertise Centre for Sexual Health, President Building, F. Rooseveltplaats 12 bus 7, 2060 Antwerpen, Belgium; eva.koppen@sensoa.be (E.K.); sandra.vandeneynde@sensoa.be (S.V.d.E.)

**Keywords:** condom use, sexual health, mixed methods, sexual risks, sexual health education

## Abstract

This study aims to address the gap in understanding condom use (CU) behavior in Flanders (the Dutch-speaking community in Belgium) by applying a mixed methods approach, integrating both quantitative and qualitative data. Utilizing a large-scale survey of over 14,000 participants and 11 in-depth interviews, the study explores key factors influencing CU, including (amongst others) relationship status, attitudes toward condoms, and STI testing. Quantitative findings highlight significant predictors such as the type of partner (casual vs. steady), STI testing behaviors, and negative attitudes toward condoms. Qualitative insights further reveal personal experiences that complicate CU decisions, such as the disruption of sexual flow and emotional dynamics within relationships. These findings underscore the complexity of CU behavior, showing that practical barriers (e.g., discomfort, fit) and personal beliefs play pivotal roles. The study concludes that targeted public health interventions could focus on improving condom accessibility and addressing both practical and emotional factors. Recommendations for sexual health education include promoting communication around CU and enhancing condom experimentation and fit. These findings contribute valuable insights into enhancing sexual health outcomes through more nuanced, contextualized approaches to condom use.

## 1. Introduction

Condom use (CU) is a critical component of public health strategies aimed at preventing sexually transmitted infections (STIs) and unintended pregnancies. Despite widespread recognition by the World Health Organization of its importance, condom use patterns and the factors influencing them remain complex and multifaceted. Research has shown that condom use is influenced by a range of factors including knowledge, attitudes, accessibility, and cultural norms [1,2,3,4]. This study aims to deepen the understanding of factors that facilitate or impede condom use across various societal and interpersonal contexts, with the goal of enhancing prevention strategies and improving overall sexual health outcomes.

### 1.1. Factors Associated with Condom Use

Research into CU behavior reveals a complex interplay of factors, including partner type, contraceptive and Pre-Exposure Prophylaxis (PrEP) use, STI testing behavior, attitudes towards condoms, knowledge, and alcohol and substance use. Various studies have examined how these factors influence the likelihood of consistent CU, with findings that often show nuanced and sometimes contradictory patterns.

Research consistently shows that CU behavior is influenced by partner type, with higher rates of consistent CU reported for casual partners compared to steady partners [5,6,7]. However, some studies found lower CU with casual partners [6]. Preparatory behaviors mediate the intention-behavior relationship for steady partners but not for casual partners [7]. Condom use with casual partners also appears to be more influenced by situational factors and partner interaction [8].

Research on contraceptive use and condom behavior reveals intricate relationships. Studies indicate that the use of hormonal contraceptives is associated with decreased CU, particularly in long-term relationships [9,10]. Factors that promote dual-use include positive attitudes towards condoms, intentions to use condoms, partner communication, and AIDS-specific counseling [11]. The perceived risk of STIs and pregnancy also influences CU alongside other contraceptives [9].

PrEP is a medication strategy used to prevent HIV infection in high-risk individuals. The impact of PrEP on CU behavior presents mixed results. Some studies indicate a significant decline in CU and an increase in STIs among PrEP users [12]. Others report that while some individuals reduced CU after starting PrEP, others maintained their previous behaviors [13]. Factors influencing CU decisions include partner familiarity and the partner’s PrEP or HIV treatment status [14]. Concerns about risk compensation and behavioral disinhibition have been raised, with some studies suggesting that PrEP users may decrease CU [15,16]. However, PrEP use has also been associated with increased confidence in discussing HIV status and CU preferences [17].

Research on the impact of STI testing on CU behavior also yields mixed results. One study found no significant changes in CU or sexual risk behaviors after HIV/STI testing and counseling [18]. However, other studies reported increased CU following testing, particularly among those who tested positive for STIs [19,20]. Factors associated with increased CU included high self-efficacy in CU, convenience of condoms, and frequent CU requests [21].

Studies on attitudes towards CU and their impact on behavior show a complex relationship. While knowledge about sexual health does not necessarily predict CU [22], attitudes toward condoms have been found to influence both past use and future intentions [23]. Negative attitudes, such as a perceived reduction in pleasure, can hinder CU [24]. Interestingly, both individuals’ own attitudes and their perceptions of their partner’s attitudes predict CU intentions and behavior [25,26]. Gender differences exist, with women generally having more favorable attitudes towards condoms [23]. However, male partners’ attitudes may exert a stronger influence on CU decisions in heterosexual relationships [27]. Day-to-day variability in attitudes, self-efficacy, and intentions also impacts CU behavior [28].

Research on condom knowledge and CU behavior presents varied results. Some studies found that increased knowledge about condoms and HIV prevention methods correlates with higher CU [29,30], while others reported no significant effect of knowledge on behavior [31]. Interventions providing information and modeling behavioral skills can enhance CU [30]. For instance, direct mailing of condom information to teenagers improved knowledge but did not significantly change attitudes or sexual behavior, although it did increase mail-order condom requests [32].

As for the association between substance use and condom use (CU), some studies found no direct link between alcohol or marijuana use and CU [33,34]. However, other research suggests that the relationship may be context-dependent. For example, alcohol consumption was associated with decreased CU during first sexual encounters [35] and among women with non-primary partners [36]. Low sexual assertiveness combined with alcohol and marijuana use increased the likelihood of risky sex among young women [37]. Notably, consistent patterns of CU or non-use were observed regardless of substance use [33]. Additionally, factors such as partner type, gender, and social class may moderate the relationship between substance use and CU [36,38].

This body of research highlights the multifaceted nature of CU behavior, influenced by partner dynamics, contraceptive methods, attitudes, knowledge, and substance use. The mixed findings underscore the complexity of sexual health behaviors and the need for tailored interventions that consider the diverse factors at play.

### 1.2. Aims of This Study

Previous studies have extensively documented the prevalence and determinants of CU in various populations. For instance, studies in European contexts have highlighted both the successes and challenges in promoting consistent CU [39,40,41]. However, much of the existing literature has relied predominantly on quantitative methods, which, while valuable, often fail to capture the nuanced experiences and personal narratives that shape individuals’ behaviors and attitudes toward CU.

In the Flemish context, there is a notable gap in the literature regarding comprehensive, mixed methods research that integrates large-scale quantitative data with in-depth qualitative insights. Such an approach is essential for understanding not just the statistical trends, but also the personal dimensions of CU. Research on CU in Flanders (the Dutch-speaking community in Belgium) is outdated [42,43], focuses on particular groups such as PreP users [44], or relies on relatively small cross-sectional samples [45]. The most comprehensive population-based sexual health study in Flanders did measure CU, yet it did not focus on contextualized experiences, enablers, and barriers [46]. This leaves a gap in understanding CU and experiences across a broader age range and diverse demographic profiles, as well as from a more in-depth qualitative perspective. Specifically, our study aims to: (1) understand and contextualize CU experiences, (2) learn about what might determine (not) using condoms, and (3) integrate these findings to inform public health strategies and interventions.

## 2. Materials and Methods

This study, commonly referred to as “The Big Condom Study”, was a collaboration between Sensoa, the Flemish expertise center for sexual health, and Ghent University. It aimed to better understand contextualized experiences, enablers, and barriers to CU in Flanders, as mandated by the Flemish government within the framework of Belgium’s federal system. In this mixed methods study, we applied a convergent design in which quantitative and qualitative methods are complementary during data collection and data analysis [47]. Moreover, the survey questions were based on the results of the exploratory interviews. This highlights that both methods are not only complementary but also mutually enriching. We will now discuss the procedure and samples of both the qualitative and quantitative studies.

### 2.1. Qualitative Study

#### 2.1.1. Procedure

Semi-structured in-depth interviews were conducted between 21 October and 19 December 2022. Participants were mainly recruited via a social media call (Facebook), and flyers were distributed near the central railway station in the largest city of Flanders. To reach hard-to-reach groups, specific calls were made, such as in a non-binary Facebook group and via snowball sampling. The call included study details and a data processing link. Interested individuals completed a short questionnaire that collected socio-demographic and personal information. Candidates who met the study criteria and whose profiles had not yet been represented in the sample were contacted for interviews, held either at a location of their choice or online via Microsoft Teams. Participants received informed consent forms to sign digitally. The interviews, conducted by the second author, lasted an average of 54 min, and participants received a EUR 40 voucher. Audio or video recordings were deleted after transcription, and transcripts were pseudonymized.

#### 2.1.2. Participants

A total of 92 individuals initially expressed interest in participating in the qualitative study. To ensure diversity, 36 candidates were contacted, with priority given to maximum variation in socio-demographic factors (age, gender, education level, migration background) and characteristics of sexual partners (gender, number, and relationship type). Participants with at least two sexual partners in the past year and recent condom use experience (within the last three months) were prioritized, providing a range of experiences for in-depth analysis. A final sample of 11 interviews was selected, aligning with qualitative research benchmarks indicating that between 9 and 17 interviews typically achieve saturation [48,49,50]. This sample size allowed us to capture a comprehensive set of themes and perspectives, with participant characteristics summarized in Table 1.

### 2.2. Quantitative Study

#### 2.2.1. Procedure

The online survey ran from 4 September to 30 November 2022, with participants recruited via self-selection. A call for participation (with a QR code or link) was shared through both online and offline media, including social media, press articles, flyers, and posters. To reduce self-selection bias, participants had a chance to win a 20 EUR voucher. Before starting, participants viewed an introduction with participation details and signed an informed consent form. The median survey completion time was 13.5 min. All data were collected via electronic forms and securely transferred to a Ghent University share, managed by authorized ICT Department staff, and were also stored on a secure server managed by Sensoa.

#### 2.2.2. Participants

The survey was accessed by 22,814 participants. Those that did not complete at least 95% of the items were removed from the sample. Furthermore, participants had to meet the following inclusion criteria: (1) being aged 16 to 80, (2) living in Flanders or Brussels or identifying as a Flemish citizen, (3) having spent sufficient time on answering the question items (i.e., at least 2 min for those who had not (yet) had sex and 5 min for sexually active participants that already had experience with external condoms). Furthermore, people who gave answers that could not be correct were excluded as well. In the end, 16,558 respondents were included in the final sample.

##### Measures

Independent variables

Control variables included sex assigned at birth (male/female), age, and financial comfort. For the latter, we posed the question, “To what extent can your family live comfortably on your total family income? By family income, we mean all the income of the people you live with, including your own. If you live alone, it refers to your personal income.” It was assessed on a five-point Likert scale ranging from “very difficult” to “very easy”.

Variables hypothesized to be associated with CU included dichotomous variables such as type of sex partners (reporting only a steady partner or also casual partners), use of contraceptives other than condoms and/or Pre-Exposure Prophylaxis (PrEP), ever having been tested for sexually transmitted infections (STIs), and having used alcohol or drugs before or during sex in the past six months. We also assessed disagreements about CU [46]. Participants indicated how often they experienced wanting to use a condom while the other person did not. There were five response options ranging from “this has never happened to me” to “this happens (almost) every time I want to have sex”, supplemented by an “I don’t remember” option. This variable was recoded into a binary variable with scores for “never or once” and “a few times to (almost) always”. Objective knowledge about condoms was assessed by presenting participants with five statements (e.g., “Condoms can be used with any type of lubricant and still function well” and “When putting on a condom, it is important to pinch the tip to leave some space at the top”) to determine whether they were correct or incorrect [31]. Slight modifications were made to make some of the items less heteronormative and less outdated. Finally, we assessed individuals’ attitudes toward CU with the Multi-Factor Attitude toward Condoms Scale (MFACS) [51]. The scale assesses perceived effectiveness (items such as “condoms are effective/not effective to prevent pregnancy”), manageability (items such as “condoms are easy/hard to obtain”), and affect (items such as “condoms increase/decrease sexual pleasure”) using a 7-point semantic differential scale (14 items in total). The scale proved sufficiently reliable (α = 0.83). Factor analysis shows that the factor structure is consistent with what was expected. Only the item “condoms are neat” did not load on any factor and was therefore removed from the analysis.

Dependent variable

Participants reporting sexual activity were asked, ‘‘In the last 6 months, how often do you use condoms when you have sexual intercourse?’’ Participants answered on a 5-point Likert scale, with the answers ‘‘rarely’’, ‘‘sometimes’’, and ‘‘usually’’ consolidated into the ‘‘sometimes’’ category. ‘‘Always’’ and ‘‘never’’ remained as the other two categories to form a three-category dependent variable.

## 3. Results

### 3.1. Qualitative Results

All transcripts from in-depth interviews were analyzed using NVivo software through Thematic Analysis [52]. This process began with familiarization with the data, followed by initial coding to highlight key statements. Researchers identified recurring patterns and organized them into themes, iteratively refining these themes to accurately represent participants’ experiences. As part of achieving reliable results, we used the principle of saturation, which, in thematic analysis, involves continuing data coding and theme refinement until no new themes or insights emerge from the data. This approach ensures that the analysis is comprehensive and that the findings reliably reflect the range of participants’ perspectives. Dewaele and Koppen thoroughly read and coded the transcripts, with Dewaele identifying 132 initial codes, which were refined to 42. Ultimately, two larger themes emerged: the condom use (CU) decision process and CU experiences, each including four subthemes: introducing the condom, negative experiences and barriers, problems related to CU, and positive experiences and enablers. This structured approach enabled a thorough exploration of participants’ perceptions and meanings, with hashtags (#) indicating the number of participants supporting a particular topic (in bold) alongside illustrative quotes.

#### 3.1.1. The Condom Use Decision Process

The decision to use a condom involves participants assessing what works best regarding contraceptive methods and condom characteristics (e.g., brands, sizes, latex or non-latex, thickness, smell, texture). It is described as **a** trade-off (#10) between high- and low-risk situations, a more pleasurable versus a more conscious encounter, the hassle of using condoms versus the availability of STI treatments, and the (dis)advantages of condoms versus other contraceptives. This also includes personal preferences and the nature of the relationship (casual or long-term). Elena explains this trade-off in relation to different sexual acts:

And in terms of safety, I never really think about that with oral sex: I can catch something from this too. Then I try to trust that the guy doesn’t have an STI, but you can’t always tell. With vaginal sex, for me it’s about safety in terms of pregnancy.

Changes (#8) in CU can be gradual (e.g., starting PrEP) or sudden (e.g., an STI diagnosis). Other reasons include STI stigma, changes in serostatus, relationship status, gender of partners, or contraceptive use. Some mentioned age (increased risk awareness) and generational differences (e.g., younger generations being less fearful of HIV). Jerome illustrates a gradual shift:

Jerome: No. I kept using condoms for a very long time, until 2018 or so. Then I went on PrEP, and my condom use dropped drastically.

Interviewer: Has that been overnight?

Jerome: That has been systematic. It started maybe 80%/20% and then quietly decreased. More and more people went on PrEP.

Many participants mentioned the straightforwardness of using condoms to avoid STIs or pregnancies. However, doubt about using condoms (#9) often arose due to a partner’s negative attitude, balancing risk versus trust and/or sexual pleasure, the desire to enhance the physical experience, running out of condoms, and doubts about their protection:

Annelies: Ultimately, when I’m with someone, I know whether I want to use condoms or not. Sometimes it’s difficult, if it’s incredibly good sex and they initiate without it, I could lose my ratio. Then you have to reason again and refuse.

Participants also assessed risk (#5) related to avoiding unplanned pregnancies or STIs. This involved considering factors such as one’s or a partner’s number of sexual partners, STI testing, use of other contraceptives, the risk of CU errors (e.g., tearing), and the type of sexual activity (e.g., oral sex without a condom).

#### 3.1.2. Condom Use Experiences

##### Introducing the Condom

Introducing a condom (#11) is a pivotal moment, which participants introduced in both direct and indirect ways. Some took the initiative, asking questions like, “Shall I put it on?” or, “Do you carry condoms with you?”. Others did it playfully, teasing the partner (e.g., dropping the condom) and using humor. Indirect methods included placing a condom in sight or referencing contraceptive use.

Several participants noted how introducing a condom could disrupt the flow (#9) of sex, marking a transition toward penetration and often leading to orgasm for one or both partners.

Annelies: One of the things I find most difficult about a condom is that it affirms the “race to orgasm”. You’re exploring with foreplay, but when penetration is coming, you’re immediately out of the “flow”. Then there’s the question: does he have his own condoms, or do I use mine? What size? What kind? It’s always a bit of a hassle.

The decision often coincides with assessing if it is the right moment and whether it risks causing a loss of erection. This is more deliberate when having multiple partners or engaging in various activities (e.g., anal and oral sex). Spontaneous sex adds complexity, whereas planned sex allows preparation and easier access to condoms.

Putting on a condom (#11) is sometimes seen as annoying, difficult, or disruptive.

Reinhard: The other agreed to use a condom, but by the time we got to that point, and I had taken the condom, the excitement had ebbed away.

Participants said it becomes easier with experience and confidence, especially with elaborate foreplay to make the activity more sensual. Opinions on who should put on the condom varied. Some women preferred to do it themselves for control or when they felt more dominant, while others let their partners do it.

Reinhard: If the partner puts it on, it’s more pleasant, especially if done playfully. When I put it on myself… You can’t tickle yourself you know?

##### Negative Experiences and Barriers

Participants refer to negative experiences with condoms that create barriers to CU, such as decreased pleasure or discomfort (e.g., a condom that feels too tight). The fuss and awkwardness of putting on a condom (e.g., finding the correct side) make users conscious of practicalities and disrupt the sexual flow, leading to a loss of connection. This can cause erectile dysfunction, creating feelings of failure. Performance pressure and insecurity can make it worse, as illustrated by Thomas:

That’s very embarrassing [losing an erection]. As a man, you feel like: I’ve failed, or something’s wrong with me. You start doubting yourself. Later, you realize it’s just stress. Knowing something is expected isn’t good for performance.

Some long-term condom users refer to “condom tiredness”. Other barriers include lack of knowledge, partners objecting to condoms, cost, alcohol influence, taboos around buying condoms, and downplaying risks. This is illustrated by Rik:

Rik: Yes, I know there are other STDs that can be more annoying. You really don’t want HIV. And other STDs? Well, I guess that’s the price you pay for having sex.

Not using a condom with a partner was also seen as symbolizing relationship closeness. Condoms are often abandoned when a partner is perceived as part of an enduring or intimate trustful relationship, as shown in this quote:

Interviewer: What would need to change for you to stop using a condom?

Julie: …An emotional connection. Not using a condom is almost like introducing your partner to your parents.

##### Problems Related to CU

When participants refer to problems with condom use, it often relates to the wrong condom size (#9):

Julie: If my bed partner doesn’t get hard enough, the condom feels loose, like a plastic bag. … I also had a partner who always bought condoms that were too big, and they would slip off. A condom shouldn’t slip off. He bought a large or extra-large when he didn’t need to.

A condom that’s too large increases the risk of slipping, while one that is too tight causes discomfort and may tear (#7). Participants stress the importance of a proper fit and exploring different condom characteristics to avoid issues. A lack of knowledge about these characteristics is seen as problematic. Slipping and tearing lead to stressful experiences, requiring STI testing or the morning-after pill, which can cause side effects:

Jennifer: One time it ripped because we used cheap condoms. That was on holiday. A friend had to get the morning-after pill. It was terrible. It was my first time taking these hormones, and it was a disaster.

Participants also mention incorrect use (#6), such as keeping condoms in wallets, opening packages with teeth, and premature or belated condom removal. A particular form of misuse is stealthing, where a partner removes the condom without consent during sex. This is seen as a terrible and transgressive act:

Jerome: I thought that was awful. I don’t understand how someone could do that. You must be a bad person. If someone says to use a condom and you take it off without consent, you have no respect.

##### Positive Experiences and Enablers

Participants refer to positive experiences (#6) with condoms that enable condom use (#11), such as making condom use a habit, reducing sensitivity for longer sex, the fact that condoms are lubricated, and the fact that they make sex more “clean” and “hygienic” (e.g., no sperm leaking from the body). This is illustrated by Leila:

Leila: But going to the toilet afterward, I’m kind of glad I don’t have to do that now.

Interviewer: You mean the hassle or the physical that it runs out?

Leila: Yes, that you can’t stay in bed and cuddle because it leaks.

The availability of condoms also enables their use, referring to always carrying them, getting them for free, or their presence at places where you have sex, as well as access to different brands and sizes.

Julie: When I lived with roommates, there was a big jar of condoms in the toilet. I was the only one that used those, but I was like: they’re there, use them if needed.

Not knowing your partner well or having sex with someone new encourages condom use. Participants refer to feelings of safety, protection, and reassurance regarding STIs and pregnancy. Some note that condom use allows multiple sex partners without risk and stress and highlights the advantage of avoiding hormonal contraceptive side effects.

Robin: I will never have sex without a condom. … Being non-binary, I prefer to avoid female hormones. … I used to take the pill, which really didn’t make me feel like myself. It made me much more emotional and depressed. The moment I stopped taking the pill, it improved. The downside is the high risk of pregnancy. Hence, always a condom.

To summarize, our qualitative results show that the CU decision process is shaped by a variety of factors, including assessments of the risks and benefits of CU, such as contraception options, condom characteristics, and situational factors (e.g., casual vs. long-term relationships). Participants described CU decisions as a trade-off between protection from STIs or pregnancy and the perceived negative aspects of condoms, such as diminished pleasure or awkwardness. Shifts in CU practices were attributed to life events or changes in personal health status, such as starting PrEP or being diagnosed with an STI, illustrating how these transitions can happen gradually or suddenly.

### 3.2. Quantitative Results

For this study, we selected only those participants that had sex during the past six months. The sample (*n* = 14,155) descriptive statistics can be found in Table 2. Table 2 includes the results of statistical tests for differences between subgroups, with significance levels (*p*-values) from ANOVA and chi-square tests, as well as effect sizes (η^2^ for continuous variables and Cramér’s V for binary variables), to indicate the magnitude of these differences. To examine the effects of our independent variables on CU, a binomial logistic regression analysis was conducted. The dependent variable was coded as “0” for never using condoms, “1” for sometimes using condoms, and “2” for always using condoms (=reference level). Independent variables included control variables (sex assigned at birth, age, and financial comfort), dichotomous variables (i.e., type of sex partners, use of contraceptives and/or PrEP, having been tested for STIs, having had arguments with partner(s) about CU, and having used alcohol or drugs prior to or during sex). The MFACS was operationalized by calculating the average score of its 13 items. This composite score was used as a continuous variable in the analysis to represent attitudes towards condoms (higher scores reflecting more positive attitudes). The level of objective knowledge was also entered as a continuous variable that reflects the number of correct answers to the five knowledge questions (range: 0–5).

Prior to the main analysis, preliminary analyses including checks for multicollinearity, distribution of variables, and missing data treatment were performed to ensure the data met the assumptions required for logistic regression. Variables were checked for linearity with the logit of the dependent variable by creating interaction terms between each continuous predictor and its natural logarithm and subsequently testing their significance. Model fit was evaluated using the -2 log-likelihood ratio test. The strength of the association between independent variables and the dependent variable was expressed in terms of odds ratios (ORs) with 95% confidence intervals (CIs). All analyses were conducted using SPSS 29 at a 0.05 significance level.

We report the results from a logistic binomial regression model comparing consistent CU (reference category) with two other categories (never used condoms and sometimes used condoms) (see Table 3).

A multinomial logistic regression was conducted to examine the predictors of CU, comparing the “Never Used” and “Sometimes Used” categories to “Always Used” as the reference group. The model was significant, χ2(22) = 4421.10, *p* < 0.001, explaining 32% of the variance in CU behavior (Nagelkerke pseudo-r-squared).

For the comparison between “Never Used” and “Always Used” condoms, several predictors were significant. Having a steady partner (compared to reporting casual partners) was associated with a higher likelihood of never using condoms. Individuals not using contraceptives and/or PrEP were significantly less likely to never use condoms compared to those not using these methods. Those who had been STI-tested (versus never tested) were more likely to report never using condoms. Negative attitudes towards condoms also significantly decreased the likelihood of CU. Additionally, individuals who reported having no arguments with their partner(s) were more likely to report never using condoms. Drug use was a significant predictor, with non-users less likely to never use condoms. Alcohol use and knowledge were not significant predictors in this category.

For the comparison between “Sometimes Used” and “Always Used” condoms, different patterns emerged. Similar to the previous comparison, individuals not using AC and/or PrEP were significantly less likely to inconsistently use condoms. STI testing increased the likelihood of inconsistent CU. Negative attitudes towards condoms were associated with a lower likelihood of inconsistent CU. Additionally, non-use of alcohol and drugs was significantly associated with a decreased likelihood of inconsistent CU. Having a steady partner (versus a casual partner), reporting arguments with partner(s) about CU, and knowledge about condoms were not associated with inconsistent CU.

## 4. Discussion

The primary aim of this study was to fill a notable gap in the literature regarding CU in Flanders by employing a comprehensive mixed methods approach. While previous studies have extensively documented the prevalence and determinants of CU, much of the research has relied on quantitative methods that may overlook the nuanced personal experiences influencing behaviors and attitudes. By integrating large-scale survey data from over 14,000 participants with 11 in-depth qualitative interviews, this study aimed to provide a more holistic understanding of CU, identifying key enablers and barriers and offering insights to inform public health strategies and interventions. The results of this study help us to understand why people use or do not use condoms. They reveal significant factors such as relationship status, attitudes toward condoms, and STI testing, while also uncovering nuanced personal experiences and barriers that influence CU decisions, such as the disruption of sexual flow and symbolic meanings within relationships. As such, our findings offer a comprehensive view of CU behaviors and the complex interplay between individual preferences and broader behavioral trends.

### 4.1. A Contextualized Understanding of Condom Use

Research shows that individuals with casual sex partners, compared to steady partners, have higher rates of consistent CU [5,6,7]. Our quantitative data supports this, and the qualitative data further revealed that CU is often abandoned in relationships that are perceived to be enduring or intimate and trustful. One participant compared discontinuing CU to introducing a partner to one’s parents, underscoring the emotional connection behind this choice. Feelings of love and attachment are known predictors of inconsistent CU [53]. Condomless sex is often viewed by couples as a way to build trust, increase intimacy, and strengthen the relationship [54], indicating that emotional dynamics play a key role in promoting or discouraging CU.

Participants using contraceptives or PrEP were less likely to either never or inconsistently use condoms, aligning with other studies [9,10,12]. The interviews revealed that participants who started using PrEP described a gradual decline in CU, with one participant specifically noting a systematic reduction, echoing findings from previous research [12,13]. However, recent studies suggest that increased STI incidence among PrEP users may stem from pre-existing risky behaviors rather than PrEP-induced risk compensation [55]. This suggests that individuals may seek PrEP during periods of heightened sexual risk, highlighting the importance of addressing risk behaviors alongside PrEP use.

Regarding STI testing, our data indicates that those who have been tested for STIs are more likely to either never or inconsistently use condoms, a finding that is somewhat contradictory to studies linking testing with more consistent CU [19,20]. Some participants from the qualitative study viewed STI testing as a way to mitigate perceived risks, a behavior consistent with existing research [56,57]. However, healthcare providers emphasize that STI testing alone, without addressing underlying risk behaviors, could be problematic [57].

Negative attitudes toward condoms were a strong predictor of never using condoms or inconsistent CU, reinforcing findings from earlier research [24,58]. The qualitative data also highlighted common barriers, such as discomfort and disruption of sexual flow. Issues such as erectile dysfunction or interruptions to sexual mood were frequently mentioned, aligning with observed negative attitudes in the quantitative data. Attitudes towards condoms can be ambivalent, with both positive and negative beliefs coexisting [59]. Targeted interventions to improve condom attitudes have proven effective in increasing use, particularly when implemented with high fidelity [58].

Participants who reported no arguments with their partners were more likely to never use condoms, though this factor did not significantly impact inconsistent use. The qualitative data showed that participants often made condom-related decisions cooperatively, emphasizing emotional connection. Research supports that joint decision-making is associated with higher CU compared to unilateral decisions or disagreements [60,61], suggesting that the absence of conflict may further reduce CU in emotionally connected relationships.

Our study found that knowledge about condoms was not a significant predictor of either never using condoms or inconsistent CU, aligning with some studies [31] but contradicting others [29,30]. Condom knowledge does not always translate into consistent use [22], with factors such as attitudes, norms, and beliefs playing a significant role [62]. Additionally, a combination of low objective knowledge and high perceived knowledge can increase the risk of not using condoms [31], highlighting the complex interplay between knowledge and behavior.

Our quantitative data suggests that non-use of alcohol is significantly associated with a decreased likelihood of inconsistent CU, supporting participants’ claims that alcohol disrupts CU by adding to the “fuss” or inconvenience. Studies have shown that alcohol consumption, particularly with casual partners, decreases CU [63], although the impact is often context-dependent, varying by situation and partner type [35]. Drug use emerged as a strong predictor of never using condoms or inconsistent CU. Consistent with previous studies, drug users—especially those using crack cocaine or injecting drugs—are more likely to engage in unprotected sex [64,65]. Substance use during sex further decreases the likelihood of CU [66]. However, CU patterns could also remain consistent regardless of substance use [33], with perceived risk and positive attitudes toward condoms also influencing behavior among drug users [67]. The strong association between lifetime substance use patterns and condom non-use underscores the need for targeted interventions addressing both drug use and sexual risk behaviors to reduce STI transmission [68].

While the quantitative data provided valuable statistical insights, the qualitative findings revealed deeper personal and contextual elements that the quantitative predictors did not capture. Participants described how condom introduction could disrupt sexual flow, causing practical challenges such as finding the right size or ensuring proper use. This disruption, often seen as a transition point between foreplay and penetration, can cause anxiety or loss of erection, echoing findings from research on condom-associated erectile difficulties [69]. Some properties of condoms seem to conflict with what people value in sexual experiences, such as being fully immersed in the moment, feeling close to one another, and being in sync or “in flow” together. Moreover, participants emphasized the importance of condom characteristics—such as size, texture, and material—influencing their experiences. These issues, such as discomfort and slippage, reflect the significance of proper condom fit [70] and underscore the need for personalized condom options [71]. Finally, the symbolic meaning of condoms emerged as an important theme, with many participants viewing condomless sex as a sign of trust and intimacy, reflecting research on the emotional significance of CU in relationships [72,73].

### 4.2. Strengths, Limitations, Suggestions for Future Research, and Practical Relevance

One of the key strengths of this study is its mixed methods approach, which combines quantitative data from over 14,000 participants with in-depth qualitative interviews. This approach enables a more comprehensive understanding of condom use (CU), integrating statistical trends with nuanced personal experiences. The large sample size adds robustness to the quantitative findings, while the qualitative component provides deeper insights into contextual factors, such as the emotional and relational dynamics influencing CU. However, the study is not without limitations. The cross-sectional nature of the data prevents us from making causal inferences about the relationships between predictors and CU behaviors. Additionally, certain variables were assessed in a binary manner (e.g., contraceptive use, drug, and alcohol consumption), potentially oversimplifying complex behaviors and missing gradients in risk.

A potential limitation lies in self-selection bias, as both the quantitative and qualitative samples may reflect participants who were more willing to discuss sensitive topics, potentially affecting generalizability. We sought to address this in the qualitative study by implementing several methodological strategies to enhance the rigor and trustworthiness of our findings. Specifically, we applied thematic analysis to systematically identify and categorize recurring themes across interviews, which helps reduce subjective bias in data interpretation. We also employed a maximum variation sampling strategy to capture a diverse range of perspectives across socio-demographic backgrounds. Nonetheless, the reliance on a convenience sample may limit the generalizability of the findings, as the sample may not represent the broader population in Flanders.

Future research should prioritize longitudinal studies to better capture changes in CU behaviors over time and explore causal relationships between factors such as STI testing, PrEP use, and condom attitudes. Expanding the assessment of variables beyond binary measures will provide a more detailed understanding of how different levels of contraceptive, drug, and alcohol use impact CU. Additionally, examining a more diverse and representative sample will help ensure that findings are applicable across various demographic and social groups.

As consistent CU is recognized as one of the most effective strategies to prevent unwanted pregnancies and sexually transmitted infections [74], this study’s findings have important implications for both sexual health education (SHE) and sexual health policy. For SHE programs, the emphasis should be placed on practical CU skills, including discussions around common barriers (e.g., loss of erection), as well as the advantages (e.g., cleanliness) of CU. Facilitating open discussions between sexual partners (e.g., about how to smoothly integrate condom use into sex: when to put it on, who puts it on, etc.) can enhance comfort and ease in sexual interactions, helping to make condom use a more natural part of the experience. On the policy side, providing a variety of condoms at low or no cost could help address issues of discomfort and fit, both of which emerged as barriers to consistent CU. Offering test kits for individuals to experiment with different condom types could further enhance comfort and satisfaction. By addressing both educational and practical barriers, interventions can better support consistent and effective CU.

## 5. Conclusions

This study provides a comprehensive exploration of CU in Flanders, combining quantitative data from a large sample with qualitative insights to offer a nuanced understanding of the factors influencing CU behaviors. The findings reveal key enablers and barriers, such as relationship status, attitudes toward condoms, and STI testing, alongside personal experiences such as the disruption of sexual flow and the symbolic meanings of condomless sex. By highlighting both the broader trends and the individual complexities of CU, this research contributes valuable insights for public health strategies. Future research should adopt longitudinal designs and consider more detailed assessments of variables such as contraceptive use and substance consumption. From a practical perspective, sexual health education programs should focus on skill-building around CU and communication within relationships. Policymakers can enhance condom accessibility by providing affordable, diverse options and allowing individuals to experiment with different condom types to improve fit and comfort.

## Figures and Tables

**Table 1:** Descriptive statistics of participants from in-depth interviews.

Pseudonym	Non-European Migration Background	Educational Level *	Age Category	Gender	Has a Steady Sex Partner	Gender of Sex Partners Last Year	Number of Sex Partners Last Year
1. Annelies	No	Higher	36–45	Female	No	Same- and opposite-sex partners	2–4
2. Elena	No	Higher	26–35	Female	Yes	Opposite-sex partners	2–4
3. Jennifer	No	Higher	26–35	Female	Yes	Opposite-sex partners	2–4
4. Julie	No	Higher	26–35	Female	Yes	Same- and opposite-sex partners	5+
5. Leila	Yes	Middle	18–25	Female	Yes	Opposite-sex partners	2–4
6. Jerome	No	Higher	46–55	Male	Yes	Same-sex partners	5+
7. Reinhard	No	Higher	46–55	Male	Yes	Same- and opposite-sex partners	5+
8. Rik	No	Higher	36–45	Male	No	Same-sex partners	2–4
9. Thomas	No	Higher	26–35	Male	No	Opposite-sex partners	2–4
10. Lucien	Yes	Middle	26–35	Non-binary	No	Same- and opposite-sex partners	5+
11. Robin	No	Middle	18–25	Non-binary	Yes	Same- and opposite-sex partners	5+

* Educational level was identified as lower (did not complete secondary education or had no formal education), middle (completed secondary education but did not pursue higher education), and higher (completed education beyond secondary level).

**Table 2:** Sample descriptive statistics.

Variable	Never Uses Condoms Mean (SD) or %	Sometimes Uses Condoms Mean (SD) or %	(Almost) Always Uses Condoms Mean (SD) or %	Total Sample Mean (SD) or %	Test of Difference	Effect Size
Sex assigned at birth (n = 14,142)					χ2 = 73.44 ***	V = 0.07 ***
Female	64.4%	56.6%	58.2%	60.1%		
Male	35.6%	43.4%	41.8%	39.9%		
Age (n = 13,870)	35.29 (12.79)	30.84 (12.07)	30.00 (10.96)	32.65 (12.26)	F = 222.76 ***	η^2^ = 0.03
Financial comfort (n = 13,870)	3.88 (0.85)	3.87 (0.87)	3.94 (0.84)	3.89 (0.85)	F = 7.67 ***	η^2^ = 0.01
Type of sex partners (n = 14,155)					χ2 = 604.99 ***	V = 0.21 ***
Steady	92.5%	74.7%	82.7%	84.0%		
Casual	7.5%	25.3%	17.3%	16.0%		
Uses AC and/or PrEP (n = 14,155)					χ2 = 1286.95 ***	V = 0.30 ***
No	20.2%	16.8%	48.4%	26.8%		
Yes	79.8%	83.2%	51.6%	73.2%		
Ever tested for STI (n = 14,015)					χ2 = 72.82 ***	V = 0.07 ***
Yes	50.4%	50.8%	42.5%	48.3%		
No	49.6%	49.2%	57.5%	49.6%		
Attitudes (n = 13,870)	2.52 (0.50)	2.60 (0.51)	2.94 (0.47)	2.66 (0.53)	F = 833.25 ***	η^2^ = 0.11
Argue with partner(s) (n = 13,758)					χ2 = 234.50 ***	V = 0.13 ***
Never or once	79.4%	66.1%	70.3%	72.5%		
A few times to (almost) always	20.6%	33.9%	29.7%	27.5%		
Knowledge (n = 13,870)	4.01 (0.99)	3.94 (1.02)	3.00 (0.98)	3.98 (1.00)	F = 7.15 ***	η^2^ = 0.01
Alcohol use (n = 14,155)					χ2 = 120.67 ***	V = 0.09 ***
No	44.4%	35.5%	46.1%	41.9%		
Yes	55.6%	64.5%	53.9%	58.1%		
Drug use (n = 14,155)					χ2 = 181.09 ***	V = 0.11 ***
No	88.7%	81.9%	91.1%	87.1%		
Yes	11.3%	18.1%	8.9%	12.9%		

Note. *** *p* < 0.001.

**Table 3:** Unstandardized regression parameter estimates, odds ratios (ORs), and 95% confidence intervals (CIs) from multinomial logistic regression of predictors of condom use.

Variable	Never Used vs. Always Used Condoms		Sometimes Used vs. Always Used Condoms	
	b (SE)	OR (95% CI)	b (SE)	OR (95% CI)
Steady partner (Ref = casual)	1.33 (0.08)	3.77 *** (3.22, 4.41)	−0.09 (0.07)	0.92 (0.81, 1.05)
Not Using AC and/or PrEP (Ref = use)	−1.60 (0.06)	0.20 *** (0.18, 0.23)	−1.61 (0.06)	0.20 *** (0.18, 0.22)
STI-testing (Ref = never tested)	0.35 (0.05)	1.41 *** (1.27, 1.57)	0.26 (0.05)	1.30 *** (1.17, 1.45)
Attitudes towards condoms	−1.92 (0.05)	0.15 *** (0.13, 0.16)	−1.34 (0.05)	0.26 *** (0.24, 0.29)
No arguments with partner(s) (Ref = having had arguments)	0.71 (0.06)	2.04 *** (1.81, 2.29)	0.05 (0.06)	1.05 (0.94, 1.17)
Knowledge	0.04 (0.03)	1.04 (0.99, 1.09)	−0.04 (0.03)	0.96 (0.91, 1.01)
No alcohol use (Ref = use)	0.01 (0.05)	1.02 (0.92, 1.12)	−0.15 (0.05)	0.86 *** (0.78, 0.95)
No drug use (Ref = use)	−0.52 (0.09)	0.60 *** (0.50, 0.71)	−0.60 (0.08)	0.55 *** (0.47, 0.64)

Note. *n* = 13,352. Nagelkerke pseudo-*R*^2^ = 0.32; model χ2(22) = 4421.10, *p* < 0.001; total classification = 56%. *** *p* < 0.001.

## Data Availability

Pseudonymized quantitative data can be requested by e-mail (alexis.dewaele@ugent.be). Due to the private nature of the qualitative data, the latter are not available to third parties.
